# Supplementary material for: Proximal tubular dysfunction as a predictor of AKI in Hospitalized COVID-19 patients
Source: PLoS One. 2024 Jun 6;19(6):e0298408. doi: 10.1371/journal.pone.0298408 (PMC11156389; doi:10.1371/journal.pone.0298408)
Supplement: S1 File — (PDF) [file pone.0298408.s002.pdf]

Operational definitions

1. Proximal tubular dysfunction: Presence of at least two of the four abnormalities – inappropriate uricosuria, renal phosphate leak, normoglycemic glycosuria and proteinuria will be labeled as PTD. Renal sodium leak and renal potassium leak were included as possible markers of proximal tubular dysfunction, but not included in the definition of PTD [1,2]. All the 6 markers were calculated based on an analysis of urine from a 24 hour urine collection in a urine pot and a same-day serum analysis.

2. Hyperuricosuria: Defined by serum uric acid (SUA) levels <220 micromol/L (3.7 mg/dL) in men and <184 micromol/L (3.1 mg/dL) in women, and a fractional excretion of urate {[(urine uric acid/SUA)/(urine creatinine/ serum creatinine)] × 100 g } > 10% [2].

3. Renal phosphate leak: Defined by a ratio of TmPi/GFR <0.77 mmol/L. This ratio was calculated in two steps as follows: (a) calculation of the renal tubular reabsorption of phosphate (RTP) with the following formula: RTP = 1 – [phosphate clearance (CPi)/creatinine clearance (Ccr)] × 100 and (b) interpretation of RTP value: if RTP $\leq$ 0.86: TmPi/GFR = RTP × plasma phosphate (Pp, mmol/L) and if RTP > 0.86: TmPi/GFR = $\alpha \times$ Pp with $\alpha = 0.3 \times \text{RTP}/[1 - (0.8 \times \text{RTP})]$ [1].
Hypophosphatamia was defined as a value under the laboratory threshold of 0.78 mmol/L or 2.4 mg/dL.

4. Normoglycemic glycosuria: was defined by a glycosuria of + on a urine dipstick test or urine routine examination (equivalent to > 15 mg/dL) and glycemia of < 180 mg/dL or 10 mmol/L. At least 1+ glycosuria as measured by dipstick in the absence of hyperglycemia (RBS < 180 mg/dl or 10 mmol/L) [1,2].

5. Proteinuria: High urinary protein creatinine ration (PCR): Proteinuria > 300 mg/g [2].

6. Renal sodium leak: Fractional excretion of sodium > 1 [3].

7. Renal potassium leak:

If normokalemia, fractional excretion of potassium > 16%

If hypokalemia, fractional excretion of potassium > 9.5%

If hyperkalemia, fractional excretion of potassium < 10% [4].

8. Acute Kidney Injury (AKI): As defined by KDIGO, An abrupt (within 48 hours) reduction in kidney function currently defined as an absolute increase in serum creatinine of more than or equal to 0.3 mg/dl (≥ 26.4 µmol/l) within 48 hours, a percentage increase in serum creatinine of more than or equal to 50% (1.5-fold from baseline) within 7 days, or a reduction in urine output (documented oliguria of less than 0.5 ml/kg per hour for more than six hours) [5].

9. Baseline serum creatinine: Lowest serum creatinine recorded in the first 48 hours of hospitalization [6].

10. Complete recovery of renal function: Decrease in serum creatinine to normal/baseline, along with improvement in urine output during hospital stay [7].

11. Partial recovery of renal function: Improvement in renal function as determined by increase in urine output & a decrease in serum creatinine but serum creatinine level still above normal/baseline at the time of discharge [7].

12. Recovery from PTD: Resolution of at least 2 markers of PTD.

References

1. Elisaf M, Rizos E, Siamopoulos K. Potassium excretion indices in the diagnostic approach to hypokalaemia. QJM. 2000;93(5):318-9.

2. Espinel CH. The FENa test. Use in the differential diagnosis of acute renal failure. JAMA. 1976;236(6):579-81.

3. Goswami S, Pahwa N, Vohra R, Raju BM. Clinical spectrum of hospital acquired acute kidney injury: A prospective study from Central India. Saudi J Kidney Dis Transpl. 2018;29(4):946-55.

4. Kormann R, Jacquot A, Alla A, Corbel A, Koszutski M, Voirin P, et al. Coronavirus disease 2019: acute Fanconi syndrome precedes acute kidney injury. Clin Kidney J. 2020;13(3):362-70.

5. Mehta RL, Kellum JA, Shah SV, Molitoris BA, Ronco C, Warnock DG, et al. Acute Kidney Injury Network: report of an initiative to improve outcomes in acute kidney injury. Crit Care. 2007;11(2):R31.

6. Werion A, Belkhir L, Perrot M, Schmit G, Aydin S, Chen Z, et al. SARS-CoV-2 Causes a Specific Dysfunction of the Kidney Proximal Tubule. Kidney Int. 2020.
